# Systematic, randomized atrial fibrillation screening using detailed phenotyping with a risk prediction model combined with patch electrocardiogram in a Swedish population aged 65 years or older: the CONSIDERING-AF trial

**DOI:** 10.1093/europace/euaf190

**Published:** 2025-08-22

**Authors:** Emelie Rakai, Farzaneh Etminani, Ninia Younan, Anton Andersson, Maria Andersson, Torbjörn Vik, Stefan Kunkel, Anna Sundin, Johan Holm, Angelo Modica, Helena M Linge, Purvee Parikh, Manish Wadhwa, Johan Engdahl, Emma Sandgren

**Affiliations:** Department of Clinical Sciences, Karolinska Institutet, Danderyd University Hospital, Entrévägen 2, 182 88 Stockholm, Sweden; Department of Medicine, Halland Hospital Varberg, Varberg, Sweden, Träslövsvägen 68, 432 81 Varberg, Sweden; Research and Innovation Centre, Region Halland, Halmstad, Sweden; Center for Applied Intelligent Systems Research in Health (CAISR Health), Halmstad University, Halmstad, Sweden; Department of Medicine, Halland Hospital Varberg, Varberg, Sweden, Träslövsvägen 68, 432 81 Varberg, Sweden; Department of Medicine, Halland Hospital Varberg, Varberg, Sweden, Träslövsvägen 68, 432 81 Varberg, Sweden; Research and Innovation Centre, Region Halland, Halmstad, Sweden; Department of Medicine, Halland Hospital Varberg, Varberg, Sweden, Träslövsvägen 68, 432 81 Varberg, Sweden; Research and Innovation Centre, Region Halland, Halmstad, Sweden; Bristol Myers Squibb AB, Solna, Sweden; Pfizer AB, Stockholm, Sweden; Pfizer AB, Stockholm, Sweden; Research and Innovation Centre, Region Halland, Halmstad, Sweden; Medical Affairs, Ambulatory Monitoring and Diagnostics, Philips, San Diego, CA, USA; Medical Affairs, Ambulatory Monitoring and Diagnostics, Philips, San Diego, CA, USA; Department of Clinical Sciences, Karolinska Institutet, Danderyd University Hospital, Entrévägen 2, 182 88 Stockholm, Sweden; Department of Cardiology, Danderyd University Hospital, Stockholm, Sweden; Department of Clinical Sciences, Karolinska Institutet, Danderyd University Hospital, Entrévägen 2, 182 88 Stockholm, Sweden; Department of Medicine, Halland Hospital Varberg, Varberg, Sweden, Träslövsvägen 68, 432 81 Varberg, Sweden

**Keywords:** Atrial fibrillation, Ischaemic stroke, Screening, Machine learning, Long-term ECG recording

## Abstract

**Aims:**

Atrial fibrillation (AF), often asymptomatic and underdiagnosed, is an independent risk factor for ischaemic stroke. A knowledge gap remains regarding the optimal target population and method to use for AF screening. We aimed to test whether screening for AF using a machine learning–based risk prediction model (RPM) and 14-day continuous patch electrocardiogram (ECG) (Philips ePatch) in high-risk individuals ≥ 65 years is more effective than standard care.

**Methods and results:**

Individuals ≥ 65 years were assigned to general or RPM cohort. The general cohort was randomized to control or invitation. In the RPM cohort, high-risk individuals, identified by RPM, were randomized to control or invitation. The primary outcome was 6-month AF incidence, analysed as intention-to-invite, comparing RPM + invitation with general + control. Of the 2960 randomized individuals, participation was 43% (632/1480) in invitation arms. Atrial fibrillation incidence was higher in RPM + invitation than in general + control arm (3.8%, 28/740 vs. 0.7%, 5/740; *P* < 0.001), yielding a risk ratio of 5.6, [95% confidence interval (2.2, 14.4)], and a number needed to invite of 32. Atrial fibrillation was more often detected in RPM + invitation than in general + invitation arm (1.1%, 8/740; *P* < 0.001), but not more often than in RPM + control arm (2.2%, 16/740; *P* = 0.07). No difference was found between general + invitation and general + control arms (1.1%, 8/740 vs. 0.7%, 5/740; *P* = 0.40).

**Conclusion:**

Among high-risk individuals ≥ 65 years, the combination of a machine learning–based RPM and long-term ECG recording was superior to standard care in identifying new AF cases.

## Graphical Abstract

**Graphical Abstract:**
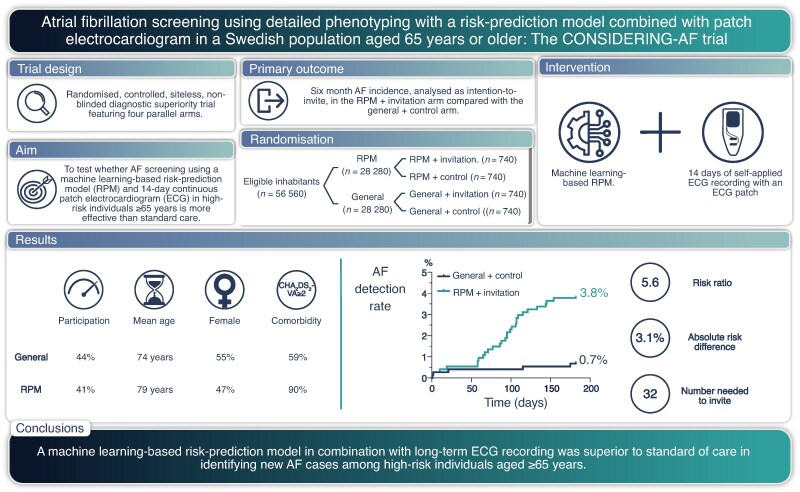

What’s new?

- The combination of a machine learning–based risk prediction model and long-term electrocardiogram (ECG) recording was superior to standard care in identifying new cases of atrial fibrillation (AF) in a population aged 65 years or older.
- Targeted AF screening may improve screening efficiency, but the benefit of identifying these AF cases requires further validation with regard to hard clinical outcomes.
- Screening high-risk individuals with 14-day long-term ECG recording leads to detection of numerous arrhythmias other than AF, but the clinical significance of these findings and their optimal management remain unclear.

## Introduction

Atrial fibrillation (AF) is the most common clinical arrhythmia, and its prevalence is steadily increasing.^1–3^ Despite the clinical burden AF imposes, AF often remains undetected due to short-lasting and asymptomatic episodes.^4^ Importantly, AF is a major risk factor for ischaemic stroke,^5^ but timely diagnosis and treatment with oral anticoagulants (OACs) can reduce the stroke risk by approximately 64%.^6^

The AF yield largely depends on the baseline prevalence of AF in the target population and the duration and method of monitoring. For instance, in the STROKESTOP trial, 2 weeks of intermittent handheld electrocardiogram (ECG) recordings in a general population aged 75–76 years resulted in a 3.0% detection rate of new AF cases^4^ and demonstrated a modest net clinical benefit.^7^ In contrast, the STROKESTOP II trial, which used N-terminal pro–B-type natriuretic peptide for enrichment, found no significant difference in stroke or systemic embolism between the screened and control groups.^8^ Similarly, the LOOP study employed implantable loop recorders in individuals aged 70–90 years with one additional stroke risk factor and achieved a 32% AF detection rate yet failed to show a reduction in stroke and systemic embolism.^9^ Additionally, opportunistic screening strategies in primary care have also not consistently outperformed standard of care.^10–12^ Currently, the 2024 European Society of Cardiology guidelines recommend considering systematic AF screening in individuals aged ≥75 years, or those aged ≥65 years with additional stroke risk factors.^13,14^

These mixed findings highlight a persistent knowledge gap regarding how to define the optimal target population and method for AF screening. Advances in digital health and access to large-scale healthcare data now enable more detailed phenotyping of individuals at high risk for AF. Recently, a validated machine learning–based risk prediction model (RPM), using routine clinical variables,^15^ has shown improved predictive performance over earlier models.^16^ Such tools can support more targeted screening strategies by identifying individuals most likely to benefit.

Simultaneously, technological advancements have expanded the availability of digital ECG monitoring devices.^17^ Among these, ECG patches offer continuous long-term ECG monitoring, making them well-suited for AF screening.^18,19^

This study combines these two approaches—machine learning–based risk stratification and long-term ECG patch monitoring—to test whether targeted AF screening in a high-risk population aged ≥65 years improves detection of incident AF compared to standard of care.

## Methods

### Trial design

The CONSIDERING-AF (deteCtiON and Stroke preventIon by moDEl scReenING for Atrial Fibrillation) trial was a randomized, controlled, siteless, non-blinded diagnostic superiority trial with four parallel arms. The trial compared targeted AF screening using a machine learning–based RPM combined with long-term ECG recording, with standard of care in a Swedish population aged ≥65 years (ClinicalTrials.gov identifier NCT05838781). The trial protocol has been previously published.^20^ The trial adhered to principles of the Declaration of Helsinki, and ethical approval for data collection from both invitation and control groups was obtained from the Swedish Ethics Review Authority (Dnr 2022-07235) and the Swedish Medical Products Agency (Dnr: 5.1-2023-18410).

### Participants

The trial was conducted in Region Halland, located in southwestern Sweden, with a population of approximately 330 000 residents. Since 2009, Region Halland has compiled the Regional Healthcare Information platform (RHIP),^21^ a comprehensive database that includes information on primary care, emergency care, outpatient specialist care, inpatient care, patient mortality, and dispensed medications from the National Prescribed Drug Register. The target population was residents in the Region Halland aged 65 years or older without a recorded diagnosis of AF in their electronic health records. Exclusion criteria included a prior diagnosis of AF, death occurring after extraction and randomization, migration outside Region Halland, the presence of a cardiac implantable electronic device, a diagnosis of dementia, or other indication for OAC treatment. Eligible individuals were randomly assigned to general and RPM cohorts. In the general cohort, a random sample was randomized to invitation or control. In the RPM cohort, the model identified high-risk individuals (RPM score > 5%), who were then randomly assigned to invitation or control.^20^

### Electrocardiogram recording procedure in invitation arms

**Figure 1:**
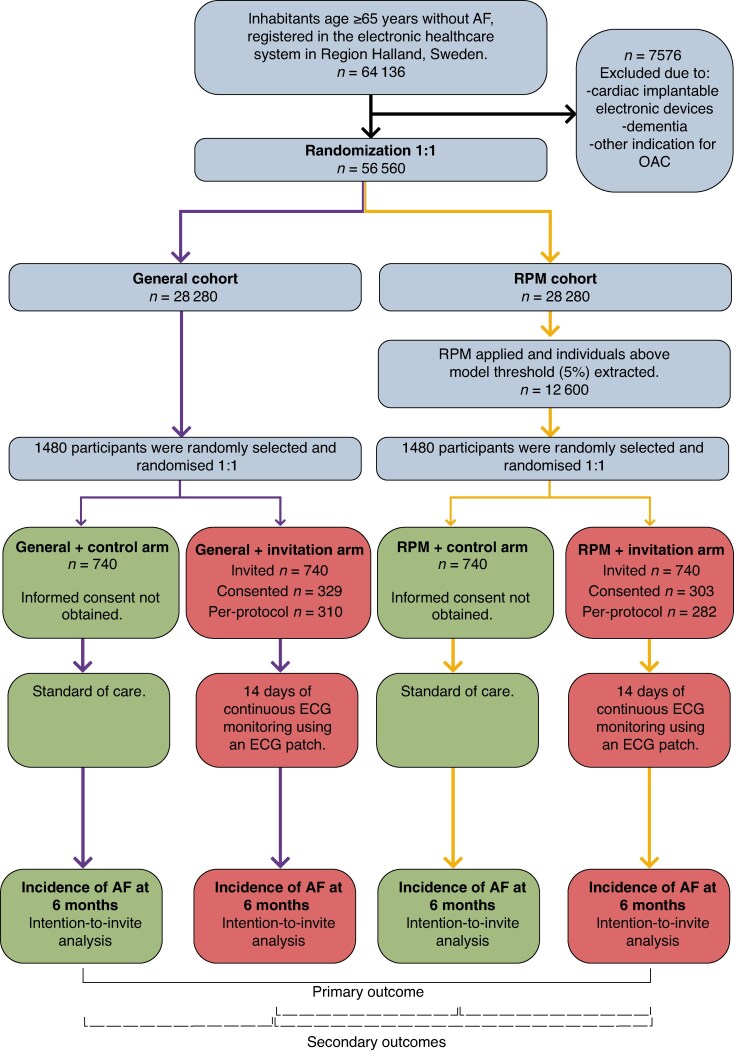
Flowchart of the study design. AF, atrial fibrillation; ECG, electrocardiogram; OAC, oral anticoagulation; RPM, risk prediction model. Flowchart adapted from Etminani *et al.* 2024, published under CC-BY license.^20^

Individuals allocated to invitation arms (*Figure 1*) were invited to undergo 14 days of ECG recording using a patch-based recorder (Philips ePatch Extended Holter, www.philips.com/epatch). Invitations were sent via postal mail, and informed consent was obtained digitally through Mobile Bank-ID (Finansiell ID-Teknik BID AB, https://www.bankid.com/en). Non-responders received up to two reminders within 2 weeks of initial invitation.

For those who consented, the ECG patch, along with instructions on how to apply the device and a prepaid return envelope, was mailed out to the participant. Participants self-applied the ECG patch, with telephone support available as needed. Upon completion of the recording period, the ECG patch was returned to the provider using the prepaid envelope.

The ECG data were uploaded by the provider to an artificial intelligence (AI)–powered ECG analysis platform (Philips Cardiologs). The ECGs were analysed in a core facility led by two cardiologists (E.S. and J.E.). Atrial fibrillation was defined as at least one episode of AF or atrial flutter lasting at least 30 s or more. All ECG diagnoses of AF were adjudicated independently by two certified cardiologists (E.S. and J.E).

Participants diagnosed with AF were contacted by telephone within 24 h, in accordance with the study protocol, to assess for initiation of OAC treatment. They were also offered an appointment with a physician. Participants with significant arrhythmias other than AF or atrial flutter (i.e. second-degree or third-degree atrioventricular block, supraventricular tachyarrhythmias, ventricular tachyarrhythmias, markedly increased proportion of premature ventricular contractions, or sinus arrest) were contacted by telephone to assess possible symptoms, and clinical follow-up was arranged according to standard of care. Participants without AF or other significant arrhythmias were informed by postal mail.

Participants allocated to control arms (general + control, RPM + control) had no contact with the investigators, and pseudonymized data were collected from routine data for standard care within the health plan organization.

### Outcomes

The primary outcome was the difference in the 6-month incidence of newly diagnosed AF or atrial flutter (in RHIP) between patients identified as at increased risk of AF through the RPM and invited for 14 days of ECG recording (RPM + invitation) and patients who received neither risk stratification nor ECG recording (general + control).

Secondary outcomes were the difference in the 6-month incidence of newly diagnosed AF or atrial flutter (in RHIP) between

1. RPM + invitation and RPM + control
2. RPM + invitation and general + invitation
3. General + invitation and RPM + control
4. General + invitation and general + control

Primary and secondary outcomes were analysed using the intention-to-invite approach. Additionally, a per-protocol analysis was performed.

Additional exploratory outcomes included the difference in proportions of patients with AF receiving OAC treatment across the four trial arms at 6 months post-randomization and the adoption rate of the self-applied ECG patch in the two invitation arms. Adoption rate was defined as the proportion of mailed out ECG patches that resulted in the upload of at least 7 days of interpretable ECG signal during the 6-month screening period.

### Data collection

Baseline patient demographic data, comorbidities, and information on dispensed medications were retrieved from RHIP after allocation.^21^ At the end of the 6-month study period, novel AF diagnoses and information on dispensed medications were retrieved from the same platform. The RHIP is updated monthly and collects data from the electronic health record system and national registers.

### Risk prediction model

The RPM was initially developed using machine learning to identify the most relevant AF risk factors from a German real-world insurance claims dataset of patients with and without known AF. The final model, based on logistic regression, consisted of 13 variables: age, sex, hypertension, heart failure, valvular heart disease, chronic kidney disease, stroke, hemiplegia, other pulmonary heart disease, paroxysmal tachycardia, other cardiac arrhythmias, ulcer of lower limb, and personal history of medical treatment.^15,20^ We calibrated the intercept (*α*) for the logistic regression using data from Region Halland. The intercept represents the baseline log-odds of an AF event prior to the contribution of individual risk factors. The 13 odds ratios for the risk factors were known from the original German study. Using a brute-force approach, all intercept values within the range [−5, 5] were tested to identify the one that best matched the event rate reported in the original German study.

### Statistical analysis

The power calculation was based on the following assumptions^1^: 6% of the screened participants in the RPM + invitation were expected to be diagnosed with AF, as indicated by data from prior AF screening studies utilizing continuous long-term ECG in individuals aged ≥ 65 years,^18,22^ while the detection rate with standard care was estimated to be 1% per year.^2^ A 50% participation rate was anticipated in the invitation groups, resulting in an estimated AF detection rate of 3.5%. This approach provided 90% power to detect a significant difference between the RPM + invitation and general + control arms, with an alpha of 0.05 and a required sample size of 737.

Randomization was carried out using a computer-generated random number sequence with a one-to-one block allocation ratio. Participants were stratified according to age and sex within both the invitation and control groups to ensure a balanced distribution of participant characteristics.

Continuous variables are presented as means ± standard deviation or median and interquartile range (IQR). Normality was assessed by the Shapiro–Wilks test. Group differences were assessed using Student’s *t*-test, Mann–Whitney *U* test, Kruskal–Wallis test, and one-way analysis of variance (ANOVA). Categorical data are reported as proportions and absolute frequencies, with differences, including the primary outcome, evaluated using *χ*² tests. The cumulative incidence of AF was evaluated using Kaplan–Meier survival curves. All tests were two-sided, with a significance threshold set at *P* < 0.05. Statistical analyses were performed using IBM SPSS Statistics version 29.0 (IBM, NY, USA) and GraphPad Prism 10.0.2 (GraphPad Inc., CA, USA).

## Results

### Participants

At the time of data extraction in November 2023, there were 56 560 eligible inhabitants, aged 65 years and older, without AF, registered in RHIP. In total 2960 participants were randomized, with 740 individuals assigned to each arm (*Figure 1*). Invitations were mailed out between 4 December 2023 and 7 February 2024. The participation rates were 44% (*n* = 329/740) in the general + invitation arm and 41% (*n* = 303/740) in the RPM + invitation arm. Among those who consented, 19 participants in the general + invitation arm and 21 in the RPM + invitation arm later withdrew their participation. Participants in the RPM arms were older (mean age ± standard deviation; 79 ± 6.7 years vs. 74.4 ± 6.7 years, *P* < 0.001) and more often male (53% vs. 45%, *P* < 0.001). Additionally, participants in the RPM arms had a higher incidence of comorbidities and more often had a CHA_2_DS_2_-VA score of ≥2 (90% vs. 59%, *P* < 0.001) compared with participants in the general arms (*Table 1*).

**Table 1:** Baseline characteristics for the four study arms

Characteristics	General + control	General + invitation	RPM + control	RPM + invitation	*P*-value
	*n* = 740	*n* = 740	*n* = 740	*n* = 740	
Demographics					
Age (years) mean ± SD	74.2 ± 6.6	74.5 ± 6.7	79.5 ± 6.7	79.5 ± 6.6	<0.00001
Age ≥ 75 years	326 (44%)	357 (48%)	575 (78%)	583 (79%)	<0.00001
Male	345 (47%)	325 (44%)	391 (53%)	392 (53%)	0.0004
Medical history					
Heart failure	20 (2.7%)	16 (2.2%)	45 (6.1%)	37 (5.0%)	0.0002
Hypertension	421 (57%)	422 (57%)	656 (89%)	630 (85%)	<0.00001
Diabetes mellitus	96 (13%)	114 (15%)	164 (22%)	145 (20%)	<0.00001
Ischaemic stroke, transient ischaemic attack, or systemic embolism	59 (8.0%)	66 (8.9%)	113 (15%)	109 (15%)	<0.00001
Coronary artery disease	77 (10%)	91 (12%)	151 (20%)	146 (20%)	<0.00001
Peripheral artery disease	26 (3.5%)	29 (3.9%)	58 (7.8%)	56 (7.6%)	0.0001
Valvular heart disease	29 (3.9%)	23 (3.1%)	51 (6.9%)	70 (9.5%)	<0.00001
Chronic kidney disease	10 (1.4%)	11 (1.5%)	43 (5.8%)	22 (3.0%)	<0.00001
Chronic obstructive pulmonary disease	40 (5.4%)	39 (5.3%)	61 (8.2%)	69 (9.3%)	0.0028
Thromboembolic risk					
CHA_2_DS_2_-VA score median (IQR)	2 (1–2)	2 (1–2)	2 (3–2)	2 (3–2)	<0.01
CHA_2_DS_2_-VA score ≥ 2	412 (56%)	459 (62%)	663 (90%)	668 (90%)	<0.00001
Antithrombotic drugs					
Oral anticoagulants	0 (0%)	0 (0%)	0 (0%)	0 (0%)	1.0
Antiplatelets	152 (21%)	172 (23%)	288 (39%)	268 (36%)	<0.00001

Reported values are *n* (%), mean ± SD, and median (IQR). Statistical tests used: *χ*² test, Kruskal–Wallis test, and one-way analysis of variance. *P* ≤ 0.05 was regarded as significant.

CHA_2_DS_2_-VA, congestive heart failure, hypertension, age > 75 years (2 points), diabetes mellitus, stroke (2 points), vascular disease, and age 65–74; IQR, interquartile range; RPM, risk prediction model; SD, standard deviation.

### Characterization of non-participants in the RPM + invitation arm

Within the RPM cohort, individuals who were randomized to invitation, but did not consent, were older and more often male and more often had a history of ulcer of lower limb compared to participants in the RPM + control and RPM + invitation arms. Additionally, they had less hypertension, less valvular heart diseases, less chronic kidney diseases, and less other cardiac arrhythmias (see *Table 2* for detailed statistics).

**Table 2:** Variables included in the RPM for participants in the RPM group

Variables	RPM + control	RPM + invitation - participants	RPM + invitation - non-participants	*P*-value
	*n* = 740	*n* = 303	*n* = 437	
Age (years) mean ± SD	79.5 ± 6.7	77.3 ± 5.3	81 ± 7.0	<0.0001
Male sex	391 (53%)	181 (60%)	369 (84%)	<0.00001
Hypertension, treated	644 (87%)	261 (86%)	350 (80%)	0.005
Heart failure, treated	51 (6.9%)	17 (5.6%)	24 (5.5%)	0.560
Valvular heart disease	51 (6.9%)	36 (12%)	35 (8.0%)	0.003
Chronic kidney disease	43 (5.8%)	9 (3.0%)	13 (3.0%)	0.003
Stroke, not specified as haemorrhage or infarction	61 (8.2%)	19 (6.3%)	38 (8.7%)	0.454
Hemiplegia	28 (3.7%)	7 (2.3%)	19 (4.3%)	0.335
Other pulmonary heart disease	6 (0.8%)	3 (1.0%)	5 (1.1%)	0.846
Paroxysmal tachycardia	17 (2.3%)	8 (2.6%)	10 (2.3%)	0.940
Other cardiac arrhythmias	45 (6.1%)	29 (9.6%)	21 (4.8%)	0.030
Ulcer of lower limb (not elsewhere classified)	33 (4.5%)	4 (1.3%)	30 (6.9%)	0.002
Personal history of medical treatment	36 (4.9%)	17 (5.6%)	19 (4.3%)	0.735

Reported values are *n* (%), mean ± SD. Statistical tests used: *χ*² test and one-way analysis of variance. *P* ≤ 0.05 was regarded as significant.

RPM, risk prediction model; SD, standard deviation.

### Primary outcome

**Figure 2:**
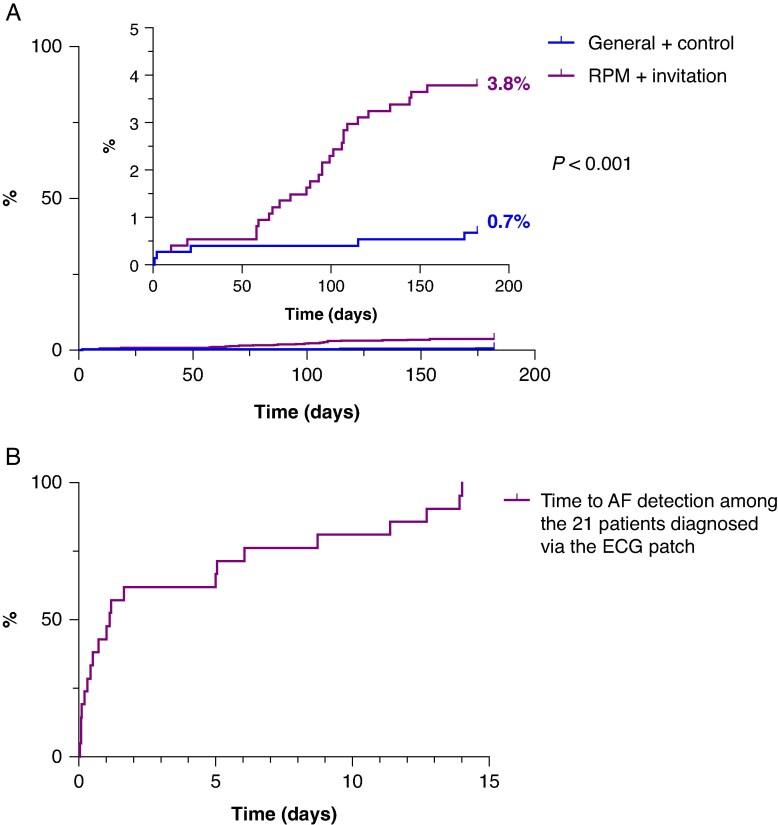
(*A*) Kaplan–Maier curve showing cumulative AF incidence in the RPM + invitation and general + control arms over the 6-month study period. (*B*) Kaplan–Maier curve showing time to AF detection during patch ECG monitoring in 21 participants diagnosed via the ECG patch. AF, atrial fibrillation; ECG, electrocardiogram; RPM, risk prediction model.

In the intention-to-invite analysis, the 6-month incidence of new AF cases was significantly higher in the in the RPM + invitation arm compared with the general + control arm (3.8%, *n* = 28/740 vs. 0.7%, *n* = 5/740; *P* < 0.001) (*Figure 2*). This resulted in a risk ratio of 5.6 [95% confidence interval (CI) (2.2, 14.4)], an absolute risk difference of 3.1% [95% CI (1.6–4.6)], and a number-needed-to-invite of 32. In the RPM + invitation arm, 16 of 28 (57%) of the AF cases were detected by the ECG patch, and 12 of 28 (42%) were diagnosed through standard of care.

### Secondary outcomes

In the intention-to-invite analysis, significantly more participants in the RPM + invitation arm were diagnosed with AF after 6 months compared with those in the general + invitation arm (3.8%, *n* = 28/740 vs. 1.1%, *n* = 8/740; *P* < 0.001). The other secondary outcomes were all non-significant: RPM + invitation arm compared with RPM + control arm (3.8%, *n* = 28/740 vs. 2.2%, *n* = 16/740; *P* = 0.07), general + invitation arm compared with RPM + control arm (1.1%; *n* = 8/740 vs. 2.2%, *n* = 16/740; *P* = 0.10), and general + invitation arm compared with general + control arm (1.1%, *n* = 8/740 vs. 0.7%, *n* = 5/740; *P* = 0.40).

In addition to new AF diagnoses, 65 participants were diagnosed with other arrhythmias (atrioventricular block, ventricular arrhythmias).

### Per-protocol analysis

In the per-protocol analysis, the 6-month incidence of new AF cases was significantly higher in the RPM + invitation arm compared with the general + control arm (6.7%, *n* = 19/282 vs. 0.7%, *n* = 5/740; *P* < 0.001). This resulted in a risk ratio of 10.0 [95% CI (3.8, 26.5)], an absolute risk difference of 6.1% [95% CI (3.1, 9.0)], and a number-needed-to-invite of 17.

In the per-protocol analysis for the secondary outcomes, the 6-month incidence of new AF cases was significantly higher in the RPM + invitation arm compared with the RPM + control arm (6.7%, *n* = 19/282 vs. 2.2%, *n* = 16/740; *P* < 0.001). Additionally, the incidence was higher in the RPM + invitation arm compared with the general + invitation arm (6.7%, *n* = 19/282 vs. 1.6%, *n* = 5/310; *P* = 0.003). No difference was seen between RPM + control and general + invitation (2.2%, *n* = 16/740 vs. 1.6%, *n* = 5/310; *P* = 0.735) and general + invitation and general + control (1.6%, *n* = 5/310 vs. 0.7%, *n* = 5/740; *P* = 0.141).

### Oral anticoagulant treatment

Among the 57 patients diagnosed with AF during the 6-month study period, 41 (72%) were prescribed anticoagulant treatment. A significantly higher proportion of patients in the invitation arms (86%, *n* = 31/36) were prescribed anticoagulant treatment compared with the control arms (48%, *n* = 10/21; *P* < 0.01).

### Feasibility of electrocardiogram patch monitoring

Out of a total of 691 ECG patches sent out, 611 (88%) were returned with at least 7 days of interpretable ECG data. The median duration of monitoring was 336 h (IQR 324–336 h), while the median time with noise was 1.9 h (IQR 0.3–16 h).

### Characteristics of atrial fibrillation detected by electrocardiogram patch

Of the 21 patients with AF diagnosed through the ECG patch during the 6-month study period, 16 (76%) patients were in the RPM + invitation arm and five (24%) patients in the general + invitation arm. Atrial fibrillation was paroxysmal in 19 (90%) patients and persistent throughout the entire recording in two (10%) patients.

The median time spent in AF was 5.2 h (IQR 0.8–26.5 h), with five (24%) patients spending less than 1 h in AF. The median AF burden was 1.6% (IQR 0.3–9.0%), and the median duration of the longest AF episode was 3.2 h (IQR 0.2–20.3 h) (*Table 3*).

**Table 3:** AF characteristics for patients with a new AF diagnosis identified during the 14-day monitoring period

	Patients
	*n* = 21
Time (h) in AF, median (IQR)	5.2 (0.8–26.5)
AF burden (%), median (IQR)	1.6% (0.3–9.0)
No of episodes, median (IQR)	1 (1–28)
Duration (h) of longest episode, median (IQR)	3.2 (0.2–20.3)
Persistent AF (%)	9.5% (2/21)
Time (days) to first detected episode, median (IQR)	1.2 (0.1–6.7)

Reported values are % (*n*/*n*) and median (IQR).

AF, atrial fibrillation; IQR, interquartile range.

The time course of AF detection is illustrated in *Figure 2*. The median time to AF detection was 1.15 days (IQR 0.1–6.7 days). Of the 21 AF cases, nine (43%) were detected within the first 24 h of recording, 16 (76%) within the first 7 days of recording, and five (24%) AF cases were detected during the second week of recording.

## Discussion

The main finding in this prospective, randomized, controlled trial was that the combination of a machine learning–based RPM and long-term ECG recording was superior to standard of care in detecting new AF cases in a population aged ≥65 years. Still the benefit of AF screening needs to be verified with regard to hard clinical outcomes.

Systematic screening for AF should be considered in patients ≥ 65 years with additional cardiovascular risk factors,^13,14^ as it has been shown to detect more new AF cases and increase the initiation of OAC therapy compared to standard care.^23–25^ However, the optimal target population and the most effective screening method remain unclear. In this trial, the combination of a machine learning–based RPM and long-term ECG recording detected significantly more new AF cases than standard of care. In the subsequent intention-to-invite analysis of secondary outcomes, the RPM alone (RPM + invitation vs. general + invitation) showed a significant contribution to the increased AF detection rate, while the long-term ECG recoding alone (RPM + invitation vs. RPM + control) did not reached statistical significance, even though there was a trend with numerical higher detection. However, the trial was not powered to detect differences in the secondary outcomes, and the lack of statistical significance of the long-term ECG recording could be attributable to a type II error. This interpretation is supported by the per-protocol analysis, which showed that both components—the RPM and the long-term ECG recording—individually contributed to a higher number of AF diagnoses. These findings suggest that both components may be important for the effectiveness of an AF screening programme.

According to per-protocol analysis, the AF detection rate was 6.7% in the RPM + invitation arm, which is higher than in the STROKESTOP (3%)^4^ and South-Norway Atrial Fibrillation Screening Study (2.2%),^26^ but lower than in the PULsE-AI study (9.4%).^27^ The higher AF detection rate compared to the STROKESTOP study may be due to higher comorbidity burden in our population and the use of continuous rather than intermittent ECG monitoring. Unlike our trial, the South-Norway Atrial Fibrillation Screening Study recruited participants via social media and daily press and had a higher proportion of female participants, which may account for the difference in comorbidity and AF detection. In contrast, the PULsE-AI study also used a machine learning–based RPM, but in a primary care setting, including individuals aged ≥30 years in the UK.^27^ Despite similar comorbidity prevalence, the higher AF detection in PULsE-AI may reflect a greater prevalence of undiagnosed AF. There have been previous AF screening activities in our region^4,28^ and the proportion of undiagnosed AF in Region Halland has been estimated to be low compared with other regions in Sweden.^29^

Although some evidence suggests that AF screening may reduce stroke-related healthcare costs,^30^ its impact on hard clinical outcomes remains debated.^31–33^ Data are conflicting regarding whether increased AF detection and subsequent initiation of OAC treatment leads to a reduction in stroke and systemic embolism.^7–9^ Advances in technology have enabled more intensive ECG monitoring, as demonstrated in our study using 14 days of continuous ECG recording, which facilitates the detection of short-lasting paroxysmal AF. However, it is likely that there is a threshold of AF burden beyond which the arrhythmia becomes clinically relevant and warrants OAC treatment.^33,34^ For instance, the STROKESTOP study used intermittent handheld ECG twice daily and showed a reduction in stroke and systemic embolism,^7^ whereas the LOOP study, which employed continuous monitoring via implantable loop recorders, failed to demonstrate a similar benefit.^9^

The current study underscores the importance of carefully selecting the target population for AF screening. The AF detection rate was low in the general ‘non-enriched’ cohort, while 42% of all newly diagnosed AF cases in the RPM + invitation arm were identified through standard of care, which means, among individuals who did not consent to the 14-day ECG recording but instead were diagnosed during standard care visits during the 6-month study period. This is likely attributable to the risk stratification achieved through the RPM, which concentrated high-risk individuals in the ‘enriched’ cohort. It is likely that the RPM cohorts had more healthcare visits than the general cohort, meaning more opportunities for AF detection as well. This is further supported by baseline data, showing a significant higher prevalence of cardiovascular comorbidities in the ‘enriched’ RPM cohort compared to the ‘non-enriched’ general cohort. Together, these findings suggest that some kind of enrichment is essential to improve the efficacy of AF screening.

Interestingly, median time to AF diagnosis with the ECG patch was 1 day and 76% of all AF diagnoses were made within the first week of monitoring, despite 90% of patients having paroxysmal AF. This may also be due to the targeting of high-risk individuals with many cardiovascular comorbidities that may have a considerable AF burden, which could indicate that these individuals are the ones most likely to benefit from OAC treatment.

Another promising approach using AI to identify individuals at high risk of incident AF is the application of deep learning–based AI to resting 12-lead ECGs.^35–37^ These methods can detect subtle, subclinical patterns associated with AF and may enable earlier identification by uncovering electrophysiological signatures not discernible to the human eye,^38^ even when using single-lead ECG recordings.^37^ However, their use depends on the availability of resting 12-lead ECG data. In contrast, the machine learning–based RPM used in this trial utilizes 13 readily available clinical parameters collected in standard care. These parameters are easily accessible through our region’s unique, comprehensive database covering healthcare and socio-demographic data for all inhabitants.^21^ This allows us to target high-risk individuals without the need for ECG data.

The participation rate was 43%, which is higher than in prior digital AF screening studies.^18,39^ In the eBrave AF trial, the participation rate was 8.2%^39^ and in the mSToPs trial 2.7%.^18^ The fact that we used postal mail, sent two reminders, and had the logotype and contact information of the local healthcare provider (Region Halland) on the invitations may have contributed to the higher participation rate. Additionally, 88% of the ECG patches sent out were returned with at least 7 days of interpretable ECG data. The median monitoring time was 336 h, with a median of 1.9 h of noise, further supporting the feasibility of using self-applied ECG patches for AF screening in older individuals.

### Clinical implications and perspective

Although the approach with targeted screening was efficient in finding patients with undiagnosed AF, the benefits of finding these patients need to be verified with regard to clinical outcomes, i.e. prevention of future AF-related stroke, heart failure, and mortality. Additionally, the health economic consequences as well as effects on healthcare utilization with a targeted AF screening programme need to be evaluated. Although AF screening has the potential to reduce future AF-related strokes, finding patients with AF is associated with a cost and at least temporarily increased healthcare consumption.

In addition, long-term ECG recording in these high-risk individuals revealed three times as many other arrhythmias than AF in the present study. These diagnoses require medical attention and mostly resulted in additional outpatient visits, which generate costs and increased healthcare utilization. The benefit of finding a rhythm abnormality and intervening before clinical presentation needs to be further explored and weighed against the risk of overdiagnosis.

### Limitations

This study has several limitations to consider. First, the RPM, originally developed by Hill *et al*.,^40,41^ was calibrated to the Region Halland population aged 65 years and older. Therefore, the applicability to a general population aged 65 years and older needs to be verified. Second, a certain amount of digital literacy was required to participate, which may have created a selection bias due to age, gender, socio-economic status, health, and digital experience. Another source for selection bias is the requirement of a Swedish Bank-ID to give informed consent. Third, the limited number of incident AF cases in the general arms introduces uncertainty in the interpretation. Fourth, inhabitants in the invitation arms, who were invited to heart rhythm monitoring, may have been prompted to be more alert to heart symptoms and accordingly more prone to seek clinical evaluation. Fifth, the effective application of an RPM on health record data presupposes a comprehensive dataset covering all relevant healthcare of the population in which screening is to be conducted; this is not available in all settings.

## Conclusion

Among individuals aged 65 years and older, a machine learning–based RPM in combination with long-term ECG recording was superior to standard of care in identifying new AF cases. Although the benefit of AF screening in regard to clinical outcomes still needs to be verified, the RPM could be a valuable tool for identifying and targeting individuals at high risk for undiagnosed AF, thereby determining who would be more effectively diagnosed with long-term ECG recording.

## Data Availability

The data underlying this article will be shared on reasonable request to the corresponding author.
